# The impact of parenting practices and family economy on psychological wellbeing and learning patterns in higher education students

**DOI:** 10.1186/s41155-024-00291-5

**Published:** 2024-03-06

**Authors:** M. A. Gandarillas, M. N. Elvira-Zorzo, M. Rodríguez-Vera

**Affiliations:** 1 Department of Social, Work and Differential Psychology, School of Psychology, Universidad Complutense de Madrid (UCM), Campus de Somosagua, Ctra. de Húmera, s/n, 28223 Pozuelo de Alarcón, Spain; 2 Department of Social Psychology and Anthropology, School of Psychology, University of Salamanca (USAL), Campus Ciudad Jardín. Avda. de la Merced 109-131, 37005 Salamanca, Spain; 3 Facultad de Odontología y Ciencias de La Rehabilitación, Universidad San Sebastián (USS), Concepción, Chile

**Keywords:** Inclusive education, Developmental psychology, Mental health, Parenting practices, Diversity in learning, Higher education

## Abstract

**Background:**

There is a large literature on the significant impact of rearing factors in the psychological development of different child’s learning patterns and wellbeing in elementary and secondary schools, but there is a scarcity of studies on to what extent those influences remain stable up to higher education.

**Objective:**

In this study, parenting practices and family status were analyzed as predictors of the different learning styles, psychological difficulties, mental health factors, and academic performance, comprising the psychosocial diversity in learning (DinL) at the university classroom.

**Methods:**

Using a cross-sectional design, a questionnaire was administered to a sample of 2522 students at the Complutense University of Madrid (Spain). It included a DinL scale measuring five psychological learning dimensions (*coping with difficulties*, *effort*, *autonomy*, *Social/Physical Context*, and *understanding/career interest*), plus several items on retrospective parenting practices, family, and sociodemographic variables. Multiple regressions and analyses of variance were conducted with the family factors as independent variables and the learning factors as dependent variables.

**Results:**

Results showed parenting variables, parents’ education, and family economy as having a significant impact on psychological learning dimensions, academic performance, and especially on the students’ wellbeing and mental health status, being an important contributors to explain the DinL in the university classroom.

**Conclusion:**

The results bring interesting conclusions for developmental and health psychologists when working with parents aimed at fostering wellbeing and learning strategies related to academic inclusion and achievement.

**Supplementary Information:**

The online version contains supplementary material available at 10.1186/s41155-024-00291-5.

## Introduction

There is a current interest in the research and applied fields on academic learning to understand the social factors that contribute to develop the diversity in learning (DinL) patterns, wellbeing, and psychological difficulties. This derives from an increasing awareness of professors and academic institutions about the need to take into account the growing psychosocial diversity in the classroom. Addressing the social factors that contribute to develop the DinL will facilitate academic inclusion, equality, wellbeing, and achievement.

Here, we use a novel integrative approach to DinL, defined as the set of psychological processes, styles, habits, difficulties, and psychosocial resources that comprise the different ways students learn in a classroom and their related family and social factors that affect them. The concept of DinL goes beyond the traditional studies on learning styles (e.g., Dunn et al., 1995; Felder & Brent, 2005; Kolb & Kolb, 2005; Leite et al., 2010; Martin et al., 2023), habits (e.g., Álvarez & Fernández, 2005), strategies (e.g., Fryer & Vermunt, 2018; Jiménez et al., 2018; Weinstein et al., 2002), and mental health and psychosocial difficulties related to learning (e.g., Del Valle et al., 2020; Zimmerman et al., 2005).

A special focus is here placed in mental health factors of university students and its relationship with DinL patterns. Different studies are pointing to increasing rates of anxiety and depression in young students in different countries (Auerbach et al., 2018; Centers for Disease Control & Prevention, 2021; Confederación Salud Mental España, 2023; Ministry of Universities, 2023). Mental health problems in higher education students are related with lower academic performance, greater functional disability, and university dropout (Bruffaerts et al., 2019; Hjorth et al., 2016). All of this is associated with psychosocial factors, cultural barriers, and structural obstacles (e.g., January et al., 2018; Lamis et al., 2016; Samaniego & Buenahora, 2016; Silva-Laya et al., 2020; Vidourek et al., 2014). Cognitive, emotional, and psychosocial difficulties in university learning have little presence in the research literature on learning styles, habits, and strategies. Incorporating them is fundamental for an inclusive concept of DinL.

DinL encompasses all the above concepts, as an interrelated learning system related to psychosocial factors, to facilitate a better understanding of the large psychosocial diversity within the current classroom. DinL is here understood not as a challenge but as a resource in class to improve collaborative learning, creativity, and cognitive flexibility, allowing the exchange of different knowledge, values, cultural background, and learning strategies, promoting higher participation in the class, and reducing university dropout (Fuentes et al., 2021; Gandarillas, 2022; Ismail & Aziz, 2019; Lu et al., 2021; Pozas et al., 2020; Rojo et al., 2021).

This concept of DinL was operationalized as a construct based on the literature on the field and on a preliminary study (see below), comprising five psychological dimensions:

- (1) *Coping with difficulties* includes the degree to which the student abates or regulates mental health difficulties related to learning, such as anxiety, irritability, discouragement, apathy, poor performance expectations, difficulties in the place of study, distortions on achievement attributions, low self-esteem, low perceived self-efficacy, and negative attitudes of the class group (Aliberti et al., 2019; Álvarez & Fernández, 2005; Batool, 2019; Chiodelli et al., 2018; Del Valle et al., 2020; Heritage et al., 2023; Khalil et al., 2020; Lew et al., 2019; MacCann et al., 2020; Matalinares et al., 2016; Morales & Pérez., 2019; Njega et al., 2019; Robledo & García, 2009; Taniguchi, 2019; Tinajero et al., 2020; Trunce et al., 2020; Weinstein et al., 2002). The studies on cognitive, emotional, or psychosocial difficulties and family/social stressors affecting learning show that these difficulties feed each other if not treated early using inclusive and integrative approaches (e.g., Asante & Andoh-Arthur, 2015; Chiodelli et al., 2018; Ibrahim et al., 2013; January et al., 2018; Lamis et al., 2016; Lew et al., 2019; Mirza et al., 2021; Samaniego & Buenahora, 2016; Santander et al., 2013; Tian-Ci Quek et al., 2019; Trunce et al., 2020).
- (2) *Effort* includes perseverance, regularity, delayed reward, control over time and situation, related to internal attribution on achievements (e.g., Correa, 2006; Mondragón et al., 2017; Muñoz, 2022; Pintrich et al., 2004; Weinstein et al., 2002), and academic achievement (Ahmad, 2019; Naz et al., 2020). Causal attributions appear as primary elements of performance and achievement motivation (Ramudo et al., 2017; Weiner, 2004) affecting the student’s expectations on their effort and are learned through past experience or personal, family, and academic conditions regarding success or failure (Barca-Lozano et al., 2019; Fernández de Mejía et al., 2015; González-Pienda et al., 2000; Ramudo et al., 2017).
- (3) *Autonomy* includes the student’s active search and integration of a variety of learning sources, the development of their own theories, and the pursuit of evidence and coherence of their theories and applications (e.g., Beltrán et al., 2006; Jiménez et al., 2018; Kolb & Kolb, 2005). High autonomy, self-determination, and self-regulation appear related to higher achievement in mathematics (León et al., 2015). An active-learning methodology allows the student to receive training according to their social moment, providing them with resources and strategies to know how to learn throughout life (Guerra et al., 2019).
- (4) *Learning by understanding and career interest* includes the attitude, intrinsic motivation, and self-efficacy to deeply understand the discipline, with the goal of good professional development (Brenner, et al., 1997; Gandarillas, 2022; Jiménez et al., 2018; Leite et al., 2010; Mondragón et al., 2017; Nielsen, 2016; Vautero & Silva, 2023). Having clear learning goals and differently adapted to the subjects to be learned may facilitate acquiring an adequate level of knowledge and skills and better academic results (Ramudo et al., 2017; Rodríguez et al., 2003), improving their motivation when the study task is found uninteresting (Valle et al., 2007) and maintaining academic commitment and involvement (Rodríguez et al., 2003).
- (5) *Social and physical context* includes the preference for studying alone vs. in group, the degree of dependence on the social context, and the preferred place of study (at home vs. in the university) (e.g., Aelenei et al., 2022; Álvarez & Fernández, 2005; Cobo-Rendón et al., 2020; Madrid et al., 2009; Mondragón et al., 2017).

These learning dimensions comprise an integrative structure of DinL which facilitates the exploration of social and family factors affecting different learning patterns, processes, and difficulties, in order to improve academic performance and to prevent psychological problems. Pinpointing such psychosocial determinants of the DinL in the classroom may enable effective personalized learning strategies that promote inclusion, achievement, and wellbeing (Fryer & Vermunt, 2018; Janson et al., 2022).

The literature in the field of social factors influencing child’s psychological development primary focuses on the relevance of child-rearing practices and parenting styles. When defining main rearing factors, the classic research literature focusses on three major dimensions (Gandarillas, 1995): *care* (including affection, warmth, and support for the child's development), *control* (including discipline and limits), and *protection*. Differences in the combinations of these three dimensions appear to affect in different ways on the development of psychological patterns related to learning. For instance, high levels of *care*, and medium levels of *contro*l and *protection* (by both parents), appear to be a good combination to foster self-esteem, autonomy, and a positive development for learning, in urban western cultures (Gandarillas, 1995).

Regarding the influence of child-rearing dimensions on the development of learning patterns, most authors point to parents’ care (warmth or support) as the most relevant positive influence and parents’ control as a negative influence in academic adjustment, autonomy, and performance in adolescents (e.g., Batool, 2019; Bully et al., 2019; Fuentes et al., 2015; Gordon & Cui, 2012; Moral et al., 2020; Njega et al., 2019; Robledo & García, 2009). A meta-analysis by Kim et al. (2020) concluded that parents’ engagement and involvement show a powerful positive impact on the student’s academic achievement and motivation. Other studies found parents’ support as significant in the student’s academic adjustment and achievement also in higher education (e.g., Dorrance et al., 2020; Walsh et al., 2023). Fass and Tubman (2002) reported positive parent-student attachment as a relevant protective factor in university students. Ji and Wang (2018) found parents’ abuse or neglect as having a relevant negative impact on cognitive flexibility, working memory, and inhibitory control ability in college students. Regarding control, there is not such an agreement as with care and support, with some authors finding parental control as having a positive impact on academic performance (e.g., Masud et al., 2019).

Another group of studies focuses on three basic parenting styles: authoritarian, permissive, and democratic. The democratic style, along with high levels of affection and support, and the promotion of autonomy with clear limits (“authoritative” style), appears as the most positive for a child’s secure development and good self-esteem (Agbaria & Mahamid, 2023; Gómez et al., 2015; Maccoby, 1992; Molina et al., 2017), preventing the development of cognitive, emotional, and behavioral problems (Cortés et al., 2014; Jaureguizar et al., 2018) and difficulties in academic performance and coexistence in the school (Fuentes et al., 2015; Gómez et al., 2014). The other parenting styles may increase the risk of anxious-ambivalent, disorganized, or avoidant attachment, leading to more vulnerability to anxiety and depression (Franco et al., 2014), traits that may affect academic performance, even at university levels (Gandarillas, 2022). Lack of discipline as well as excessively rigid discipline, little expression of affection, and overprotection limit the development of the child’s autonomy and personal competence, increasing the risk to depression and anxiety (Affrunti & Ginsburg, 2012; Franco et al., 2014; Gfellner & Córdoba, 2020; Hernesniemi et al., 2017; Maccoby, 1992), very relevant in the establishment of academic difficulties.

Other important family and social factors pointed out by the literature as influencing DinL are the family socio-economic level (Gandarillas, 2011; Guterman & Neuman, 2018; Rodríguez-Hernández et al., 2020; Kim et al., 2020; Martineli et al., 2018; Piccolo et al., 2016), the parents’ level of education (Guterman & Neuman, 2018; Han et al., 2023; Kim et al., 2020; Masud et al., 2019; Silva-Laya et al., 2020), the gender of the student (Bully et al., 2019; Taniguchi, 2019), and the culture and origin of the family (Kim et al., 2020; Worrell, 2014). We should also consider that family cultures, structures, and roles have undergone a deep change in the last decades, increasing the diversity in types of families and parenting styles (Cowan & Cowan, 2019; Kim et al., 2020). Therefore, we may expect an important increase and relevance of the DinL in the classroom.

The large body of studies showing the significant influences of parenting practices and family conditions on the development of learning patterns, and wellbeing of the students, is centered mostly on primary and secondary educational levels. There is not so much knowledge on to what extent these influences in the students’ learning patterns and wellbeing related to their studies stay present even at higher education levels and contribute to explain the DinL at the university classroom. Research on this topic has theoretical and applied implications, as the academic institutions and professors need to understand the DinL and its causes, to be able to address them at all educational levels. Therefore, the objective of this study is to analyze the influence of rearing dimensions and family conditions in the wellbeing and DinL in higher education. Here, it is stated as a general hypothesis that basic parenting dimensions (*care*, *control*, and *protection*) and family features (family economy and parental educational levels) will predict the DinL and mental health levels related to the studies in the university.

## Method

### Sample

The sample was composed of 2522 students (1856 undergraduate, 452 master’s, and 214 PhD students) in 85 different programs (social, science, humanities, and technical) at the Complutense University of Madrid (UCM), a public university in Spain. The mean age was 24.2 years old (standard deviation 8.4). A total of 27% of the students were men, and 73% were women. A total of 75.2% of students were born in Spain, and 24.8% were born in a large variety of countries from Africa, Asia, Europe, and the Americas.

### Instrument

DinL scale is an individually self-administered questionnaire to assess the main dimensions that define DinL in the classroom (see Additional file 1). See Gandarillas (2022) for a detailed description of the procedure used to build the scale. The 4-point (1 = nothing or very little, 2 = some, 3 = quite a lot, 4 = a lot) Likert-type scale comprises 28 items, representing 5 dimensions: *coping with difficulties* (9 items), *effort* (6 items), *autonomy* (5 items), *understanding and career interest* (5 items), and *social and physical context* (3 items). The scale showed adequate indices of adjustment for the five subscales of the model in a preliminary analysis of validation. The reliability of the scales in terms of internal consistency scores of coefficient omega was between *ω* = 0.62 and 0.80. The model fit of the scale was *CFI* = 0.932, *TLI* = 0.925, *RMSEA* = 0.065, and *SRMR* = 0.067 showing adjustment to the five-dimensions model. Additionally, eight items from the *Egma Minnen av Bardndosnauppforstran* (EMBU) scale (Arrindell et al., 1988, 2006) measured the rearing *care*, *control*, and *protection* dimensions. EMBU is a retrospective 4-points Likert-type questionnaire, widely used in different countries with optimal metric properties of the items as interval variables comprising the three major child-rearing dimensions (named by the authors as warmth, rejection, and protection) (e.g., Cheng & Wu, 2021; Mathieu et al., 2020; Yongmei & Jiaying, 2022). Two items measuring the care dimension, one item of control, and one item of protection for mother and father were included, as being optimal representations of these dimensions (Gandarillas, 1995; Gandarillas et al., 2005). The students were asked to score on these items recalling the parenting practices used to them when they were between the ages of 13 and 17 years old. In the questionnaire, educational levels of father and mother, family economic levels, academic performance (average grades on last year), and four sociodemographic variables (sex, age, nationality, and field of study) were also included (see Additional file 1).

### Design and procedure

Using a cross-sectional design, the final questionnaire including the abovementioned items was administered to a sample of 2.737 students. Participation in the study was voluntary, confidential, and anonymous, with the informed consent. This work followed ethical procedures in accordance with the Declaration of Helsinki (Word Medical Association, 2013) and had the approval of the Research Ethical Committee at the Complutense University of Madrid (ref nº CE_20211118-15_SOC).

### Data analysis

A first study of the dataset rejected all cases with more than 5% of missing data or incorrect answering (random answering or clear errors), with a final sample of 2522 valid cases for further analysis. Then, descriptive analyses were conducted to characterize the sample, assessing the mean, standard deviation, asymmetry, and kurtosis of the items. The indices of asymmetry and kurtosis showed values ± 1.96 to assume a normal distribution (Mardia, 1970). The values of inverse items (see Additional file 1) were switched (e.g., 1 = 4, 4 = 1). Factor scores of the five DinL dimensions were obtained based on an OBLIMIN oblique five-factor analysis. The internal consistency of the data was assessed through the Omega coefficient of McDonald (ω), considering a lower limit of 0.70 to get an acceptable reliability (Taber, 2018). The Pearson correlation coefficient was obtained to assess the relationships between variables and to identify the presence of multicollinearity.

To estimate the predictive value of the parenting dimensions in the DinL factors and academic performance, linear multiple regressions with the forward stepwise method were carried out. Assumptions of linearity, normality, homoscedasticity, and multicollinearity were analyzed. The criterion variables were extracted from the DinL factor scores of the DinL dimensions (regression method). The items on mother’s and father’s *care*, *protection*, and c*ontrol* were used as independent variable (IV) predictors. The mean of the two *care* items (per mother and father) was used as one variable in the regression. The *R*^2^ determination coefficient, the non-standardized coefficient (*B*), standardized coefficients (*β*), VIF indices, and tolerance were also obtained. To further validate the significant results of the multiple regressions, analyses of variance (ANOVAs) with the significant predictors of the multiple regressions (*p* < 0.05) on DinL were carried out, using the median to divide the predictive items in two levels of the IVs (low and high) and the factor scores of the DinL dimensions as dependent variables (DVs).

Besides, father’s and mother’s educational and family economic levels were used as predictors and the DinL factors and academic performance as DVs in multiple regressions, using same methods and parameters as above. ANOVAs with the significant predictors were also in this case carried out to further validate the significant results of the multiple regressions. One-way ANOVAs were carried out on the differences on each of the items measuring learning difficulties (items belonging to the *coping with difficulties* dimension), and academic performance levels (DVs) on the family economic levels (IV), to achieve a deeper understanding of the special findings regarding the group with the highest economic levels. Data analysis was conducted with the computer programs SPSS (version 27) and Jamovi project (2022) version 2.3.21.

## Results

The results in Table 1 shows the descriptive statistics (mean, standard deviation, skewness, and kurtosis) of the parenting variables and DinL factors. The skewness and kurtosis are smaller than ± 1.96, considered normally distributed (Mardia, 1970). Reliability showed adequate scores (*ω* = 0.66 to 0.79) except in the dimension *understanding/career interest* (*ω* = 0.59), perhaps due to the wider scope of the content. Table 1 also shows the correlations matrix between parenting dimensions and DinL factors, with correlations ranging between 0.05 and 0.57, without the presence of multicollinearity.

**Table 1:** Correlations between DinL dimensions, rearing variables, parents’ educational levels, family economy and academic performance in the university sample

	1	2	3	4	5	6	7	8	9	10	11	12	13	14	15
1. *Coping with difficulties*	–														
2. DinL *effort*	0.19**	–													
3. DinL *autonomy*	− .08**	0.15**	–												
4. DinL *understanding/career interest*	0.23**	0.23**	0.20**	–											
5. DinL *Social/Physical Context*	− .07**	− .09**	0.10**	.01	–										
6. Father’s *care*	.09**	0.11**	− .05*	.06**	− .07**	–									
7. Mother’s *care*	0.20**	.08**	− .09**	.09**	− .01	0.56**	–								
8. Father’s *control*	− 0.19**	-.07**	.09**	− .07**	.02	− 0.21**	− 0.19**	–							
9. Mother’s *control*	− 0.21**	− 0.11**	0.12**	− .06**	.05*	− 0.18**	− 0.21**	0.56**	–						
10. Father’s *protection*	− 0.17**	− .01	0.10**	− .03	.01	− .03	− 0.13**	0.35**	0.26**	–					
11. Mother’s *protection*	− 0.12**	− .02	.09**	.03	.04*	− .08**	.03	0.23**	0.33**	0.57**	–				
12.Father’s educational level	.01	− .07**	− .07**	.03	.04	0.30**	0.22**	.01	− .02	− .02	− .04*	–			
13. Mother’s educational level	.04	− .08**	− 0.04	.03	.09**	0.19**	0.32**	0.01	.01	− .04*	− .01	0.55**	–		
14. Family’s economic levels	0.11**	.00	− 0.10**	.03	− .00	0.24**	0.29**	− .07**	− 0.9**	− .05*	− .01	0.37**	0.39**	–	
15. Academic performance	0.14**	0.24**	0.14*	0.20**	− 0.17*	.06**	.05**	− .05**	− .05*	.01	− .04	.03	.01	.06**	–
ω	0.79	0.72	0.72	0.59	0.66										
M	0.00	− 0.01	0.00	0.00	− 0.01	2.77	2.56	1.62	1.63	2.16	2.00	2.38	2.36	2.99	3.00
SD	1.00	1.00	1.00	0.99	1.00	0.99	1.03	0.92	0.94	1.07	1.01	0.70	0.71	0.88	0.76
Asymmetry	− 0.10	− 0.03	0.13	− 0.63	0.77	− 0.35	− 0.11	1.33	1.32	0.42	0.64	− 0.68	− 0.63	− 0.04	− 0.31
Kurtosis	− 0.56	− 0.54	− 0.41	0.55	− 0.06	− 1.07	− 1.24	0.61	0.57	− 1.09	− 0.75	− 0.74	− 0.80	0.16	0.05

^**^p < .01, *p < .05. *M*, mean, *SD* standard deviation, *DinL* diversity in learning

Table 2 shows the results with the significant (*p* < 0.05) multiple regressions of parenting variables predicting the following DinL factors. Of the five dimensions that make up the DinL, coping with difficulties (the dimension approaching the mental health status regarding the student’s learning) was the factor most related to the parenting variables, where mother’s care was the predictor that most contributes to the model, followed by mother’s control and to a lesser degree father’s protection [*R*^2^ = 0.08, *F*(4, 2.309) = 52.84, *p* < 0.001]. The other four factors showed lower *R*^2^, although the coefficients got higher significant levels in all cases: effort [*R*^2^ = 0.02, *F*(2, 2.311) = 24.17, *p* < 0.001]; autonomy [*R*^2^ = 0.02, *F*(3, 2.310) = 18.45, *p* < 0.001], Social/Physical Context [*R*^2^ = 0.01, *F*(3, 2.310) = 7.22, *p* < 0.001], and understanding and career interest [*R*^2^ = 0.01, *F*(2, 2.311) = 11.67, *p* < 0.001] (see Table 2). Academic performance was predicted by father’s *care* and *control* [*R*^2^ = 0.005, *F*(2, 2.456) = 6.579, *p* = 0.001]. In all these multiple regressions, the VIF and the tolerance indices allow the rejection of collinearity of the variables (see Table 2).

**Table 2:** Multiple regressions. Rearing variables of mother and father significantly predicting (*p* < .05) DinL factors

Model	B			S.E	β		*t*		*p*	Tolerance	VIF
***DinL coping with difficulties***											
Constant		− 0.14		0.08			− 1.74		.082		
Mother’s control		− 0.13		0.03	− 0.12		− 5.05		.001	0.68	1.47
Mother’s care		0.15		0.02	0.15		7.30		.001	0.95	1.05
Father’s protection		− 0.09		0.02	− 0.10		− 4.46		.001	0.87	1.16
Father’s control		− 0.07		0.03	− .06		− 2.49		.013	0.64	1.56
***DinL effort***											
Constant			− 0.08	0.08		− 0.99		0.322			
Mother’s control			− 0.11	0.02	− 0.10	− 4.74		.001		0.97	1.03
Father’s care			0.09	0.02	.09	4.17		.001		0.97	1.03
***DinL autonomy***											
Constant		− 0.15		0.08			− 1.88	.060			
Mother’s control		0.10		0.02	.09		4.37	.001		0.91	1.10
Father’s protection		0.06		0.02	.07		3.16	.002		0.93	1.08
Mother’s care		− 0.06		0.02	− .06		− 0.28	.006		0.95	1.05
***DinL understanding and career interest***											
Constant		0.10		0.07			1.41	0.160			
Mother’s care		0.08		0.02	.08		3.66	.001		0.97	1.03
Father’s control		− 0.06		0.02	− .05		− 2.40	.016		0.97	1.03
***DinL Social/Physical Context***											
Constant		0.03		0.08			0.40	0.690			
Father’s care		− 0.10		0.03	− 0.10		− 3.88	.001		0.68	1.47
Mother’s care		0.06		0.03	.06		2.36	.018		0.67	1.49
Mother’s control		0.05		0.02	.05		2.27	.023		0.95	1.05
***DinL academic performance***										Tolerance	VIF
Constant		2.922		0.06			50.16	.000			
Father’s care		0.038		0.02	.050		2.40	.016		0.97	1.04
Father’s control		− 0.036		0.02	− .044		− 2.14	.032		0.97	1.04

*SE* standard error, *DinL* diversity in learning

Table 3 shows a series of one-way ANOVA tests to further validate the relationship of the 16 significant predictors of parenting variables grouped in low and high scores, divided by the median (as IVs) on the DinL factors and academic performance (as DVs). The results showed significant differences (*p* < 0.05) in all DinL factors and academic performance excepting those of mother’s *care* in *Social/Physical Context*.

**Table 3:** Significant (*p* < .05) one-factor analyses of variance (ANOVAs) of DinL factors and academic performance by the rearing significant predictors (grouped in low and high) in the multiple regressions

Rearing	DinL and academic performance	Rearing levels	M	SD	df	F	*p*
Father’s care	Effort	Low	− 0.13	1.00	1	30.47	.001
		High	0.10	1.00			
	Social/Physical Context	Low	0.07	1.05	1	12.34	.001
		High	− 0.07	1.00			
	Academic performance	Low	2.92	0.77	1	8.112	.004
		High	3.01	0.75			
Mother’s care	Coping with difficulties	Low	− 0.16	1.02	1	68.28	.001
		High	0.18	0.95			
	Autonomy	Low	0.60	1.00	1	11.18	.001
		High	− 0.80	1.00			
	Understanding/career interest	Low	− 0.07	0.94	1	12.25	.001
		High	0.08	1.00			
Father’s control	Coping with difficulties	Low	0.13	1.00	1	69.01	.001
		High	− 0.22	1.00			
	Understanding/career interest	Low	0.05	1.00	1	10.64	.001
		High	− 0.08	1.00			
Mother’s control	Coping with difficulties	Low	0.13	1.00	1	69.31	.001
		High	− 0.22	1.02			
	Autonomy	Low	− 0.08	1.00	1	24.05	.001
		High	0.12	1.00			
	Effort	Low	0.64	1.00	1	21.45	.001
		High	− 0.13	1.01			
	Social/Physical Context	Low	− 0.05	1.01	1	9.45	.001
		High	0.08	1.00			
Father’s protection	Coping with difficulties	Low	0.10	1.00	1	49.01	.001
		High	− 0.20	1.00			
	Autonomy	Low	− 0.07	1.00	1	18.58	.001
		High	0.12	1.01			

*M* mean, *SD* standard deviation, *df* degrees of freedom, *DinL* diversity in learning

The multiple regressions with father’s and mother’s educational levels and family economy predicting the DinL factors and academic performance showed high significant levels (with *p* < 0.01) in the following: *coping with difficulties* significantly predicted by family economic levels [*R*^2^ = 0.012, *F*(1, 2.317) = 28.99, *p* < 0.001]; *autonomy* significantly predicted by family economic levels [*R*^2^ = 0.011, *F*(1, 2.317) = 24.51, *p* < 0.001]; effort significantly predicted by mother’s educational levels [*R*^2^ = 0.006, *F*(1, 2.317) = 13.61, *p* < 0.001]; *Social/Physical Context* significantly predicted by mother’s educational levels and family economy [*R*^2^ = 0.008, *F*(2, 2.317) = 11.11, *p* < 0.001]; and academic performance significantly predicted by family economic levels [*R*^2^ = 0.003, *F*(1, 2.458) = 7.95, *p* = 0.005]. All predictions had positive direction, excepting family economy predicting Social/Physical Context. There were not significant predictions to *understanding/career interest*. In all these multiple regressions, the VIF and the tolerance indices allow the rejection of collinearity of the variables.

Table 4 shows the significant results to further validate the relationship of the seven significant predictors of parenting variables grouped in low and high scores, divided by the median (as IVs) on the DinL factors and academic performance (as DVs).

**Table 4:** Significant results (*p* < .05) of one-factor analyses of variance (ANOVAs) of DinL factors and academic performance by mother’s and father’s education and family economy significant predictors (in the multiple regressions)

	**DinL dimension**	**Educational levels**	**M**	**SD**	**df**	**F**	***p***
Mother’s education	Effort	Low	0.17	1.00	2	7.23	.001
		Middle	0.01	0.99			
		High	− 0.07	1.00			
	Social/Physical Context	Low	− 0.14	0.95	2	9.70	.000
		Middle	− 0.73	0.98			
		High	0.09	1.03			
		**Family economy**	**M**	**SD**	**df**	**F**	***p***
Family economy	Coping with difficulties	Low	− 0.35	1.18	4	11.91	.000
		Low-middle	− 0.21	1.05			
		Middle	0.03	0.96			
		High-middle	0.15	0.98			
		High	− 0.25	1.04			
	Autonomy	Low	0.57	0.99	4	11.93	.000
		Low-middle	0.12	1.03			
		Middle	− 0.06	0.98			
		High-middle	− 0.04	0.97			
		High	− 0.05	1.07			
	Academic performance	Low	2.84	0.88	4	2.83	.020
		Low-middle	2.92	0.73			
		Middle	2.97	0.75			
		High-middle	3.05	0.76			
		High	2.91	0.85			

*M* mean, *SD* standard deviation, *df* degrees of freedom, *DinL* diversity in learning

**Fig. 1:**
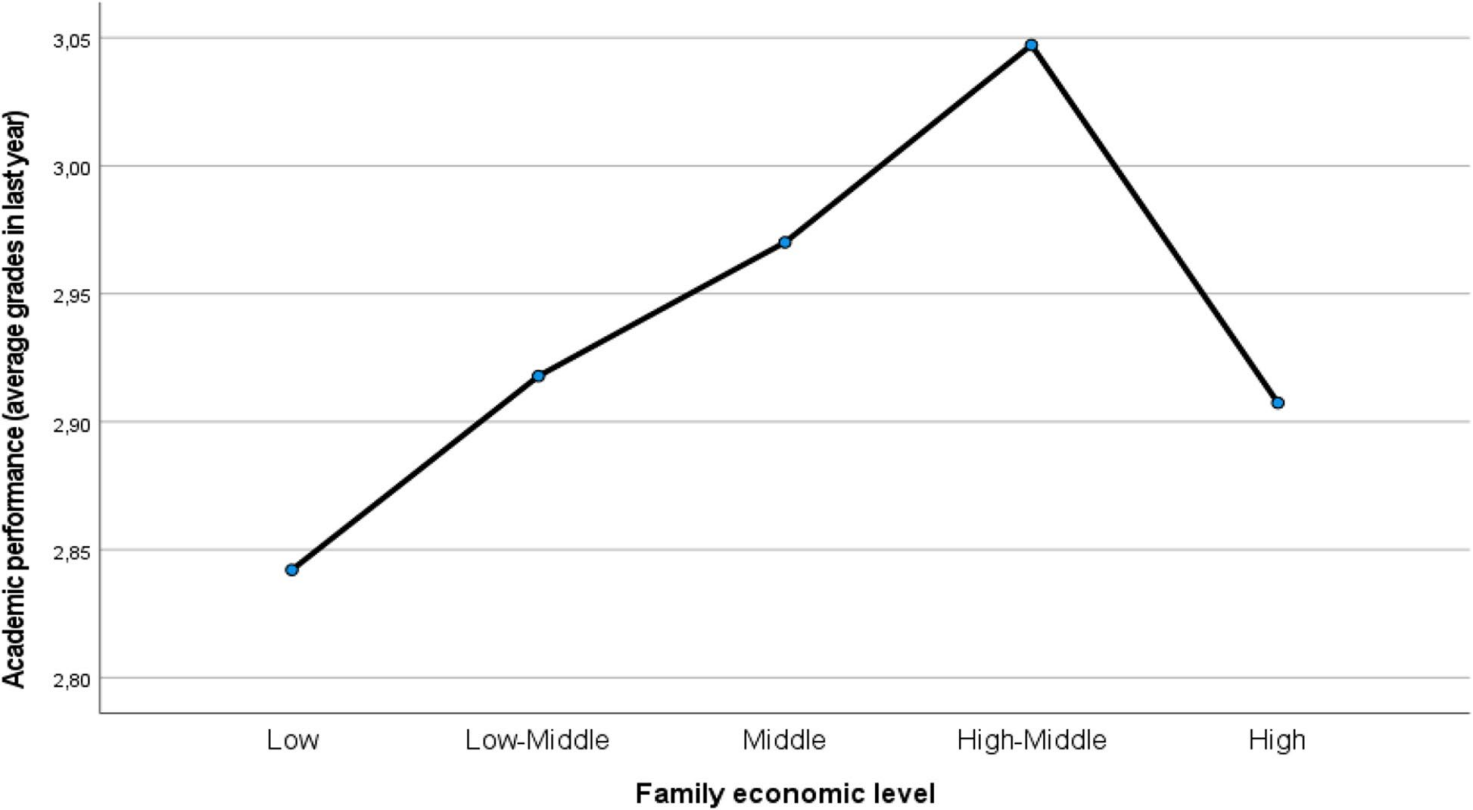
Academic performance (average grades last year) by family economic levels

Figure 1 shows the means of the academic performance levels according to the family economic levels.

**Fig. 2:**
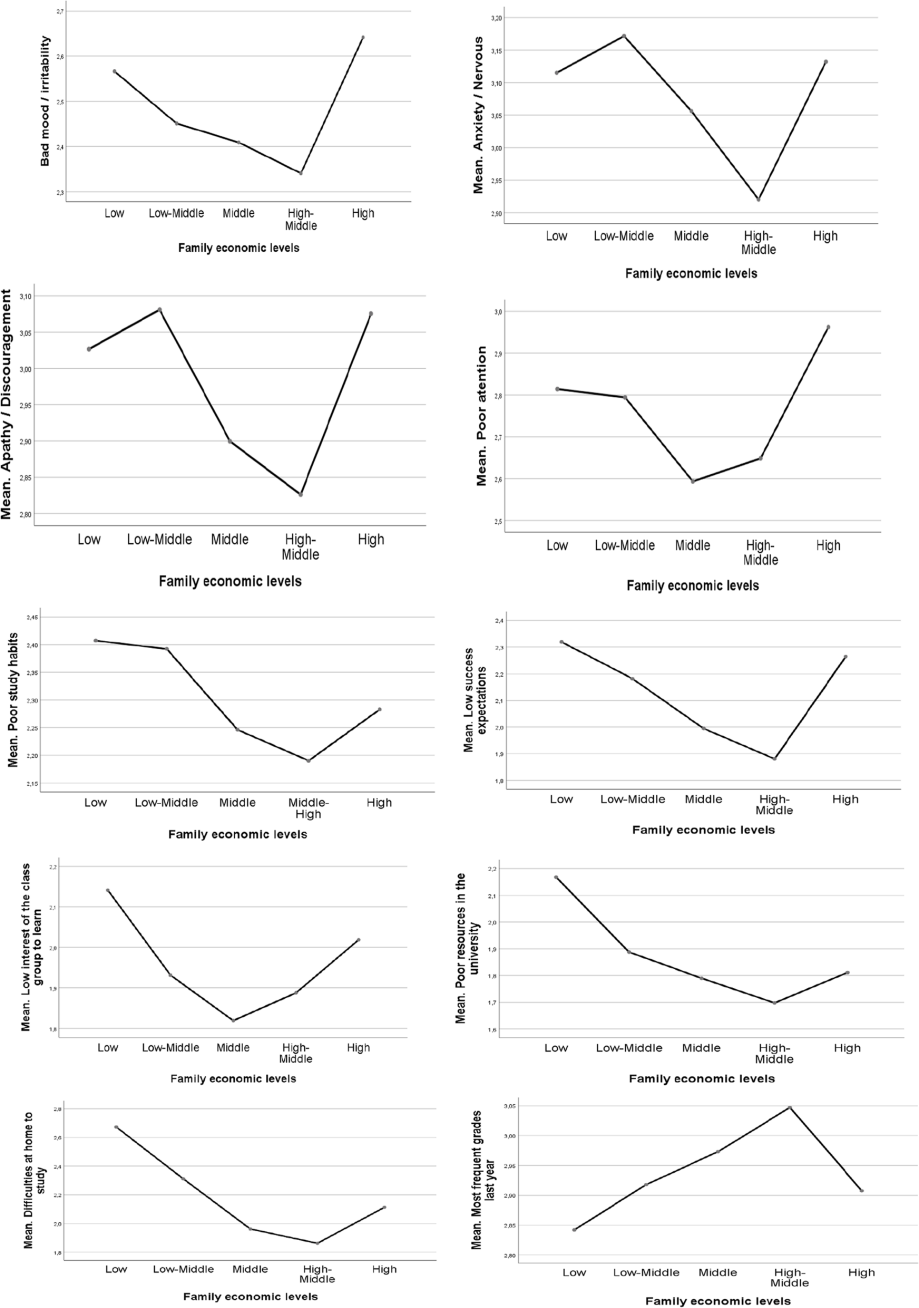
Learning difficulties (1 = nothing or very little, 2 = some, 3 = quite a lot, 4 = a lot) and academic performance (1 = F, 2 = E-D-C, 3 = B, 4 = A) by family economic levels. Significant results (*p* < .05) of analyses of variance (ANOVAs)

To further analyze the special results regarding the differences on the *coping with difficulties* factor and academic performance according to family economic levels (especially regarding the changing trend in high economic families), one-way ANOVAs were carried out with each of the items addressing learning difficulties (comprising the *coping with difficulties* dimension) and academic performance by the family economic levels, with the following results: bad mood/irritability (*F*(4, 2.454) = 2.06, *p* = 0.084); anxiety/nervous (*F*(4, 2.454) = 3.86, *p* = 0.004); apathy/discouragement (*F*(4, 2.454) = 4.26, *p* = 0.004); poor attention (*F*(4, 2.454) = 4.44, *p* = 0.001); poor study habits (*F*(4, 2.454) = 2.60, *p* = 0.035); low success expectations (*F*(4, 2.454) = 8.16, *p* = 0.000); low interest of the class group to learn (*F*(4, 2.454) = 4.01, *p* = 0.003); poor resources in the university (*F*(4, 2.454) = 7.98, *p* = 0.000); difficulties at home (*F*(4, 2.454) = 21.85, *p* = 0.004); and academic performance (*F*(4, 2.467) = 2.80, *p* = 0.025). Figure 2 shows the means of the items with significant results according to the levels of family economy. Only the item regarding bad mood/irritability did not show significant results (*p* < 0.05).

## Discussion

In general terms, the results supported the main hypothesis. The main parenting practices *care*, *control*, and *protection* showed significant predictions and differences on all the DinL factors. Next, we analyze the results according to the DinL psychological dimensions as follows:

### Coping with psychosocial difficulties

This dimension comprises the mental health and wellbeing indicators related to learning and appears as the most affected learning dimension by the perceived father’s and mother’s parenting patterns and family economic levels. Mother’s *care* and *control* appear as the most important predictors of psychosocial difficulties (anxiety, lack of motivation, poor attention…) but in opposite directions. Maternal control and discipline seem to increase difficulties in studying and learning, in line with other studies in the field. Gómez et al. (2015) found maternal support as the main factor to foster coping with difficulties in studying and possible higher resilience. Gandarillas (2011) reported inhibited physiological reactions to punishment in college students with higher levels of maternal care and involvement during childhood (implying a protective factor under academic anxiety). In this study, the role of the father seems to be less relevant in predicting *coping with difficulties*, with father’s *control* and *protection* appearing with a significant negative impact in developing coping abilities. These results on the influences of mother’s and father’s rearing patterns reflect (at the university level) the main studies done at different educational levels (Batool, 2019; Bully et al., 2019; Franco et al., 2014; Fuentes et al., 2015; Gómez et al., 2015; Gordon & Cui, 2012; Kim et al., 2020; Masud et al., 2019; Moral et al., 2020; Njega et al., 2019; Robledo & García, 2009; Taniguchi, 2019).

### Effort

Results suggest that lower mother’s control and higher father’s support led to a higher emphasis on using effort as a resource to study. Again, higher control as a parenting practice appears as a negative factor, supporting different studies in the field (Affrunti & Ginsburg, 2012; Batool, 2019; Bully et al., 2019; Franco et al., 2014; Gandarillas, 2022; Masud et al., 2019; Robledo & García, 2009).

### Autonomy

Here, the results are somewhat startling. Those university students with higher levels of autonomy report lower maternal *care*, higher mother’s *control* and father’s *protection* (in secondary education), which seems to refute classical findings (e.g., see Maccoby, 1992; Gandarillas, 1995). However, taking into consideration that even the high father’s *control* and *protection* levels in the sample (see Table 3) are within a middle range, we might not expect very hard, authoritarian control practices in the students scoring higher control and protection, but possibly closer to “authoritative” styles. The combining results may suggest that the students developing higher autonomy in their studies tend to opt for searching learning resources by themselves when their parents dedicate less support to their studies but promote responsibility and self-discipline. These results partially support research which points to a democratic and supportive parenting style (promoting freedom but supervising the limits) to boost students’ autonomy in their studies (Cortés et al., 2014; Fuentes et al., 2015; Gómez et al., 2014, 2015; Maccoby, 1992). The promotion of the student’s autonomy and active participation in their learning process, being capable of making decisions in their own learning process, is a main goal underlined in the European Higher Education Area (Delors, 1998). This study corroborates the family as a significant resource to contribute to this goal.

### Understanding and career interest

Results point to higher mother’s *care* and lower father’s *control* (before the university) as the significant predictors of learning by understanding in the university aimed at getting the needed competences of the professional role. These parenting patterns may also be linked to the parents’ trust in their child’s capacities to successfully accomplish their career (Šimunović & Babarović, 2020) and the family qualities to foster concept and deep learning as an academic strategy (Brenner, et al., 1997; Nielsen, 2016).

### Social/physical context

Lower father’s *care* and higher mother’s *control* (before the university stage) seem to lead to a higher preference to study in group outside the home and in the university. Mother’s *care* appears also as a significant predictor, but it is not backed by the results of the ANOVAS. It is worth noting that the results are opposite to those on the dimension *effort*, with which it shows a significant negative relationship (see Table 2). Students preferring studying at home (without their classmates) may also have developed higher effort-based skills, promoted by their parenting styles.

The low *R*^2^ shown on several multiple regressions may limit the extent of the inferences on some of these results. This may be due to the high DinL of the student’s sample which leads to a higher dispersion of the data. However, the consistent pattern of high significant levels in those regressions combined with the high significant differences in the ANOVAs on the same variables gives additional validity to the inferences here.

Regarding academic performance results provide further support to the previous results. As Tables 2, 3 show, academic performance is significantly predicted especially by father’s care (before the university studies) in a positive relationship and by father’s control, in a negative relationship (although control did not reach significant levels in the ANOVA). Again, this supports the relevance of the impact of care and (in less extent) control, in this case on the learning outcomes.

In general terms, when focusing on the mother’s and father’s role in the development of the different dimensions of the DinL, the relevant role of the mother is underlined. Mother’s *care* and *control* appear as significant predictors of all the DinL dimensions. The father’s role, when it appears as significant, sometimes shows a negative impact (as in the case of *coping with difficulties*) although in other moments it appears as powerful (as in *effort*, *autonomy*, and academic performance). The results suggest that when the father is present and fully involved in the child’s learning, the impact is very important.

Here, it is worth noting that even though the mother’s dimensions are more significant than the father’s on the development of DinL, the only parenting variable without any significant prediction is mother’s *protection*. These results as a whole suggest that the traditional father’s and mother’s roles — the mother having the main caring role and the father a main protective role (Carlson & Knoester, 2011; Graf & Wojnicka, 2023; Gregory & Milner, 2011) — are still present, at least regarding the development of the student’s learning dimensions in this sample.

The results regarding mother’s and father’s education and family economical levels provide additional support to our results. Again, the role of the mother appears especially relevant. Higher mother’s educational levels appear significantly related to a higher *effort* and to place more relevance to the *Social/Physical Context* in the study. In terms of perceived family economic levels, the results are highly relevant. Higher family’s economic levels show stronger coping with learning difficulties (such as anxiety, demotivation, low attentional levels…), in line with other studies (e.g., Martineli, 2018; Rodríguez-Hernández et al., 2020; Silva-Laya et al, 2020). The results on academic performance also support these findings. Also, lower family economy appears related to higher *autonomy* of the students, which may partly explain the mother’s difficulties to provide higher support to their children in their studies in families with lower income (Tables 3, 4). The positive relationship between family economy levels and the tendency to study at home (although not backed by the ANOVA results) may also give further support to this an explanation. Finally, the significant positive relationship between family economic levels and academic performance (Table 4 and Fig. 1) point again to the great relevance of the family economy in the student’s learning patterns and their impact on their academic achievement, as shown in different studies (Gandarillas, 2011; Guterman & Neuman, 2018; Rodríguez-Hernández et al., 2020; Piccolo et al., 2016; Silva-Laya et al., 2020).

An interesting exception is found in the results of the group with the highest family economical levels, which turns the trends, showing lower coping levels facing almost all the psychosocial difficulties in learning included in this study and showing poorer academic performance than middle and high-middle economic levels (Table 4, Figs. 1 and 2). These families may have higher expectations about the student’s grades, which may lead to higher stress and negative results on academic performance. It might also suggest being the result of a permissive (vs. democratic or authoritarian) parenting style (Franco et al., 2014; Gómez et al., 2014) in these families contributing to making the students more prone to learning vulnerabilities and low motivation, perhaps due to a difficulty in the internalization of norms.

## Conclusions

As general conclusions, the results of this study show that family features as parenting patterns and family economy have an important impact on main students’ learning dimensions and particularly on their mental health status. Main results highlight the following points:

- In most of students’ families (most of them Spanish in this sample), father and mother continue to have traditional roles with a significant impact on the development of the student’s habits, styles, strategies, and difficulties in learning, including at the level of higher education.
- The mother seems to take a more relevant role than the father in giving support to the child’s studies. Maternal care/support appears as the most important positive, protective influence when coping with mental health problems regarding the student’s learning process.
- The father appears more absent in the student’s learning development, excepting in the protecting role. The father appears having a relevant impact when being more present. For instance, high father’s care appears significantly related to higher student’s effort in their studying.
- In general terms, high levels of parental control appear as having important negative impacts on the mental health status of the students and on the levels of effort and understanding in their studies.
- Parental educational levels and family economic levels also significantly contribute to the development of the DinL in the classroom. They have relevant positive impacts on the development of students’ learning dimensions, mental health, and academic performance, excepting on the students with highest family economic levels.

As mentioned above, a large body of studies already showed the relevant influence of different rearing practices and parenting styles on the children’s learning patterns, contributing to the DinL in the classroom. This study points out that this influence on the development of the student’s learning habits, styles, strategies, and difficulties remains stable, being present also at higher education.

### Implications in the applied field

Considering the results obtained, it is important to take into account parenting practices and family characteristics in order to better understand the psychological development of the different learning patterns to improve their adaptation to the university context. The present study suggests that those learning characteristics are not so changed nor adapted by the student to the university requirements, but dragged from primary and secondary education when the student develops stable habits, styles, and strategies on how to learn, partially based on the parent’s and family dynamics. The relevance of rearing factors and parenting styles on the mental health and wellbeing of the students highlights how important working with parents and families at earlier ages is key to promote the student’s optimal academic performance up to higher education. That parents can significantly contribute to strengthen their children’s wellbeing regarding their academic performance is especially positive and hopeful in times of increasing mental health problems in youth in different countries (Centers for Disease Control & Prevention, 2021; Confederación Salud Mental España, 2023; Ministry of Universities, 2023). This study shows that family features significantly contribute to the DinL in a university classroom.

Using the integrated, psychosocial DinL approach here used, including family and social factors, professors may be able to better tailor and use diversity as a resource to improve inclusion, equality, and a rich collaborative learning. Also, secondary professors and parents can promote the adequate combination of parenting and psychoeducational patterns to develop the optimal learning strategies to prepare the student for better performance at the university level. Furthermore, this study shows the importance of studying parenting styles to prevent psychological difficulties in learning and mental health problems in university students. Finally, the results on parent’s educational levels and family economy point to the deep impact that family status still has on educational inequality, which stress again the long way that we still have to reach equality of opportunities in the educational and labor realms, at least in the society of the sample studied.

### Limitations of the study

The use of retrospective methods to report parenting practices might imply some biases (e.g., distortion of recall by current mood states, or subjective/emotional representations of fathers and mothers). However, different studies do not confirm these biases in this type of retrospective instruments, but they show stability of the recall about their parenting over the time (Gerlsma et al., 1994; Koutra et al., 2022; Richter & Eisemann, 2001; Wilhelm et al., 2005). Besides, in this study, the student’s perceptions of their parenting were more interesting than the actual parenting during childhood, as it provides more direct information on the impact of those parenting styles on the student.

*Understanding/career interest* showed lower omega values than the rest of DinL dimensions in the sample of this study. This could be due to the content of this DinL dimension, wider than the rest of the learning dimensions (therefore leading to a lower Omega).

### Future lines of research

Parenting practices and family status contribute to a proportion of DinL in the university classroom, but there are other important psychosocial factors that may also explain the DinL relevant to analyze further, such as the influence of student’s gender, the family’s culture or country, the influence of the educators’ teaching styles, or the impact of digital technologies. Finally, the research team of this study is working to gather samples at universities in other countries to increase the external validity of the results.

## Supplementary Information

**Additional file 1.** Diversity in learning (DinL) scale items grouped in the five dimensions that define it.

## Data Availability

The datasets used and/or analyzed during the current study are available from the corresponding author on reasonable request.

## Abbreviations

ANOVA: Analysis of variance
DinL: Diversity in learning
DV: Dependent variable
EMBU: Egma Minnen av Bardndosnauppforstran
IV: Independent variable
M: Mean
SD: Standard deviation
SE: Standard error
