# Correction: The impact of parenting practices and family economy on psychological wellbeing and learning patterns in higher education students

**DOI:** 10.1186/s41155-024-00333-y

**Published:** 2024-12-16

**Authors:** M. A. Gandarillas, M. N. Elvira‑Zorzo, M. Rodríguez‑Vera

**Affiliations:** 1 Department of Social, Work and Diferential Psychology, School of Psychology, Universidad Complutense de Madrid (UCM), Campus de Somosagua, Ctra. de Húmera, s/n, 28223 Pozuelo de Alarcón, Spain; 2 Department of Social Psychology and Anthropology, School of Psychology, University of Salamanca (USAL), Campus Ciudad Jardín. Avda. de la Merced 109‑131, 37005 Salamanca, Spain; 3 Facultad de Odontología y Ciencias de La Rehabilitación, Universidad San Sebastián (USS), Concepción, Chile

**Correction: Psicol. Refl. Crít. 37, 8 (2024)**

**https://doi.org/10.1186/s41155-024-00291-5**

Following publication of the original article (Gandarillas et al., 2024), the authors reported that in the Method section, the sample number by fields of study “(856 undergraduate, 452 master's, and 288 PhD students)” is incorrect. It should be replaced by the following text: 1856 undergraduate, 452 Master, and 214 PhD students.

In addition, the following statistical information in the results section is incorrect due a typing error (i.e., the information of the Fs, the commas that separate the degrees of freedom between groups and within groups were mistakenly moved one digit to the right).

- The text has been corrected:

From: Table 2 shows the results with the significant (p < 0.05) multiple regressions of parenting variables predicting the following DinL factors. Of the five dimensions that make up the DinL, coping with difficulties (the dimension approaching the mental health status regarding the student’s learning) was the factor most related to the parenting variables, where mother’s care was the predictor that most contributes to the model, followed by mother’s control and to a lesser degree father’s protection [R2 = 0.08, F(42,309) = 52.84, p < 0.001]. The other four factors showed lower R2, although the coefficients got higher significant levels in all cases: effort [R2 = 0.02, F(22,311) = 24.17, p < 0.001]; autonomy [R2 = 0.02, F(32,310) = 18.45, p < 0.001], Social/Physical Context [R2 = 0.01, F(32,310) = 7.22, p < 0.001], and understanding and career interest [R2 = 0.01, F(22,311) = 11.67, p < 0.001] (see Table 2). Academic performance was predicted by father’s care and control [R2 = 0.005, F(22,456) = 6.579, p = 0.001]. In all these multiple regressions, the VIF and the tolerance indices allow the rejection of collinearity of the variables (see Table 2).

To: Table 2 shows the results with the significant (p < 0.05) multiple regressions of parenting variables predicting the following DinL factors. Of the five dimensions that make up the DinL, coping with difficulties (the dimension approaching the mental health status regarding the student’s learning) was the factor most related to the parenting variables, where mother’s care was the predictor that most contributes to the model, followed by mother’s control and to a lesser degree father’s protection [R2 = 0.08, F(4, 2.309) = 52.84, p < 0.001]. The other four factors showed lower R2, although the coefficients got higher significant levels in all cases: effort [R2 = 0.02, F(2, 2.311) = 24.17, p < 0.001]; autonomy [R2 = 0.02, F(3, 2.310) = 18.45, p < 0.001], Social/Physical Context [R2 = 0.01, F(3, 2.310) = 7.22, p < 0.001], and understanding and career interest [R2 = 0.01, F(2, 2.311) = 11.67, p < 0.001] (see Table 2). Academic performance was predicted by father’s care and control [R2 = 0.005, F(2, 2.456) = 6.579, p = 0.001]. In all these multiple regressions, the VIF and the tolerance indices allow the rejection of collinearity of the variables (see Table 2).

- The text has been corrected:

From: The multiple regressions with father’s and mother’s educational levels and family economy predicting the DinL factors and academic performance showed high significant levels (with p < 0.01) in the following: coping with difficulties significantly predicted by family economic levels [R2 = 0.012, F(12,317) = 28.99, p < 0.001]; autonomy significantly predicted by family economic levels [R2 = 0.011, F(12,317) = 24.51, p < 0.001]; effort significantly predicted by mother’s educational levels [R2 = 0.006, F(12,317) = 13.61, p < 0.001]; Social/Physical Context significantly predicted by mother’s educational levels and family economy [R2 = 0.008, F(22,317) = 11.11, p < 0.001]; and academic performance significantly predicted by family economic levels [R2 = 0.003, F(12,458) = 7.95, p = 0.005]. All predictions had positive direction, excepting family economy predicting Social/Physical Context. There were not significant predictions to understanding/career interest. In all these multiple regressions, the VIF and the tolerance indices allow the rejection of collinearity of the variables.

To: The multiple regressions with father’s and mother’s educational levels and family economy predicting the DinL factors and academic performance showed high significant levels (with p < 0.01) in the following: coping with difficulties significantly predicted by family economic levels [R2 = 0.012, F(1, 2.317) = 28.99, p < 0.001]; autonomy significantly predicted by family economic levels [R2 = 0.011, F(1, 2.317) = 24.51, p < 0.001]; effort significantly predicted by mother’s educational levels [R2 = 0.006, F(1, 2.317) = 13.61, p < 0.001]; Social/Physical Context significantly predicted by mother’s educational levels and family economy [R2 = 0.008, F(2, 2.317) = 11.11, p < 0.001]; and academic performance significantly predicted by family economic levels [R2 = 0.003, F(1, 2.458) = 7.95, p = 0.005]. All predictions had positive direction, excepting family economy predicting Social/Physical Context. There were not significant predictions to understanding/career interest. In all these multiple regressions, the VIF and the tolerance indices allow the rejection of collinearity of the variables.

- The text has been corrected:

From: To further analyze the special results regarding the differences on the coping with difficulties factor and academic performance according to family economic levels (especially regarding the changing trend in high economic families), one-way ANOVAs were carried out with each of the items addressing learning difficulties (comprising the coping with difficulties dimension) and academic performance by the family economic levels, with the following results: bad mood/irritability (F(42,454) = 2.06, p = 0.084); anxiety/nervous (F(42,454) = 3.86, p = 0.004); apathy/discouragement (F(42,454) = 4.26, p = 0.004); poor attention (F(42,454) = 4.44, p = 0.001); poor study habits (F(42,454) = 2.60, p = 0.035); low success expectations (F(42,454) = 8.16, p = 0.000); low interest of the class group to learn (F(42,454) = 4.01, p = 0.003); poor resources in the university (F(42,454) = 7.98, p = 0.000); difficulties at home (F(42,454) = 21.85, p = 0.004); and academic performance (F(42,467) = 2.80, p = 0.025). Figure 2 shows the means of the items with significant results according to the levels of family economy. Only the item regarding bad mood/irritability did not show significant results (p < 0.05).

To: To further analyze the special results regarding the differences on the coping with difficulties factor and academic performance according to family economic levels (especially regarding the changing trend in high economic families), one-way ANOVAs were carried out with each of the items addressing learning difficulties (comprising the coping with difficulties dimension) and academic performance by the family economic levels, with the following results: bad mood/irritability (F(4, 2.454) = 2.06, p = 0.084); anxiety/nervous (F(4, 2.454) = 3.86, p = 0.004); apathy/discouragement (F(4, 2.454) = 4.26, p = 0.004); poor attention (F(4, 2.454) = 4.44, p = 0.001); poor study habits (F(4, 2.454) = 2.60, p = 0.035); low success expectations (F(4, 2.454) = 8.16, p = 0.000); low interest of the class group to learn (F(4, 2.454) = 4.01, p = 0.003); poor resources in the university (F(4, 2.454) = 7.98, p = 0.000); difficulties at home (F(4, 2.454) = 21.85, p = 0.004); and academic performance (F(4, 2.467) = 2.80, p = 0.025). Figure 2 shows the means of the items with significant results according to the levels of family economy. Only the item regarding bad mood/irritability did not show significant results (p < 0.05).

The original article (Gandarillas et al., 2024) has been updated.
