# Supplementary material for: The impact of parenting practices and family economy on psychological wellbeing and learning patterns in higher education students
Source: Psicol Reflex Crit. 2024 Mar 6;37:8. doi: 10.1186/s41155-024-00291-5 (PMC10917719; doi:10.1186/s41155-024-00291-5)
Supplement: Supplementary file 1 — Additional file 1. Diversity in learning (DinL) scale items grouped in the five dimensions that define it. [file 41155_2024_291_MOESM1_ESM.docx]

**Additional file 1**

Diversity in learning (DinL) scale items grouped in the five dimensions that define it

| Dimension | Item |  |
| --- | --- | --- |
| Coping with difficulties | **15** | The circumstances determine the final results of my studies (learning, grades), whether they are good or bad |
|  | **20** | Bad mood/ Irritability |
|  | **21** | Anxiety / nervous |
|  | **22** | Apathy/ Discouragement / Reluctance |
|  | **23** | Difficulties in attention and concentration |
|  | **25** | Poor expectations of academic achievement or success |
|  | **26** | Low interest of classmates in learning |
|  | **27** | The university lacks resources for the students |
|  | **28** | Difficulties at home in concentrating on studying (home environment, space to study…) |
| Effort | 1 | I study with perseverance and regularity |
|  | **3** | Above all, I study and concentrate under the pressure of an upcoming exam |
|  | 9 | I am able to manage my time and study environment |
|  | 10 | I am able to delay the satisfaction of desires or impulses |
|  | 12 | Daily reading frequency (including all sorts of readings) |
|  | **24** | Little consistency in my study habits |
| Autonomy | 4 | I like to develop my own theories and I pay attention to whether real examples support or refute my theories |
|  | 5 | I search for useful applications and a practical understanding of new knowledge |
|  | 8 | I read complementary readings and watch videos that are not required for the exams, for my own knowledge |
|  | 13 | I organize and integrate information gathered from different sources in my learning |
|  | 14 | I search for evidence of my theories |
| Understanding /  Career Interest | 6 | When I study I focus on understanding the concepts more than anything else |
|  | **7** | I memorize the concepts and theories without needing to understand everything perfectly |
|  | 11 | When studying, I focus primarily on relating ideas and concepts |
|  | 18 | Above all, the main focus of my studies is professional development for my career |
|  | 19 | I know all the profiles and professional prospects of my study to be able to plan my career |
| Social / Physical Context | 2 | I believe that when studying in a group I resolve questions that I cannot resolve by myself |
|  | 16 | I study in spaces at the University |
|  | **17** | I study at home |

The numbers in bold font indicate inverse items
